# Finding the Best Lighting Mode for Daguerreotype, Ambrotype, and Tintype Photographs and Their Deterioration on the Cruse Scanner Based on Selected Methods

**DOI:** 10.3390/s23042303

**Published:** 2023-02-18

**Authors:** Zuzana Berger Haladová, Róbert Bohdal, Zuzana Černeková, Paula Štancelová, Andrej Ferko, Janka Blaško Križanová, Jana Hojstričová, Kitti Baráthová

**Affiliations:** 1 Faculty of Mathematics Physics and Informatics, Comenius University, Mlynska Dolina, 842 48 Bratislava, Slovakia; 2 Conservation and Restoration Department, Academy of Fine Arts and Design in Bratislava, Hviezdoslavovo Námestie 18, 814 37 Bratislava, Slovakia

**Keywords:** CRUSE, ambrotype, daguerreotype, tintype, scanning modes, eye-tracking, concurrent color channels, sharpness function, image differences, image similarity, UIQI, SSIM, ESSIM

## Abstract

In the digital heritage field, the accurate reproduction of hard-to-photograph items, such as daguerreotypes, ambrotypes, and tintypes, is an ongoing challenge. Industrial contactless sensors offer the potential to improve the quality of scanned images, but their capabilities and limitations have not been fully explored. In our research, a dataset of 48 scans was created using the hi-tech industrial contactless sensor CRUSE. Moreover, 3 rare original photographs were scanned in 16 different modes, the most suitable images were determined by specialists in the restoration, and validated through experiments involving eye-tracking, multiple computer vision, and image processing methods. Our study identified the Cruse scanning modes, which can be utilized to achieve the most accurate digital representation of scanned originals. Secondly, we proposed several methods for highlighting the degradation and minor scratches on photographs that otherwise might not be detected by the restorer’s naked eye. Our findings belong to four overlapping areas, i.e., image understanding, digital heritage, information visualization, and industrial sensors research. We publish the entire dataset under the CC BY-NC 4.0 license. The CRUSE sensor shows promise as a tool for improving the quality of scanned images of difficult-to-photograph items. Further research is necessary to fully understand its capabilities and limitations in this context.

## 1. Introduction

The digitization of historical ambrotypes, daguerreotypes, and other photographic materials is incredibly important. First, it allows us to preserve these fragile and delicate items for future generations. Without digitization, many of these items would be at risk of being lost or damaged due to age and environmental factors.

Second, digitization makes these items more accessible to researchers and the general public. Instead of having to visit a physical archive or museum, people can now view these photographs online, making it easier for them to study and appreciate them.

Third, digitization allows us to enhance and improve these photographs. With the use of specialized software, we can restore damaged or faded images, bringing them back to life and making them more legible. We can also make high-resolution copies of these photographs, which can be used for research and educational purposes.

Overall, the digitization of historical ambrotypes, daguerreotypes, and other photographic materials is essential for preserving our cultural heritage and making it more accessible to the public. It allows us to protect these items for future generations and unlock the valuable information and stories they contain. Digitization facilitates the preservation of original heritage objects and provides a digital equivalent available for further study. This is especially important in information-bearing artifacts and paper-based artifacts. The emphasis on digitization may lead to the misconception that information matters, whereas the physical preservation of the artifact does not [1]. Digitization is not preservation. However, digital copies of paper documents may be used for further material analysis protecting valuable originals from handling.

Virtualization of cultural or natural heritage has become an important part of the knowledge economy. We create the digital twins using the hi-tech CRUSE CS 220ST1100 contactless scanner, providing high precision both in geometry and radiometry. We report on our “multiple ground truths” findings in the context of the digitization of hundreds of thousands of museum originals (cultural objects) in Slovakia.

We experimented with sharpening, scanning mode selection, and properties of scans. In this paper, we offer a detailed description of the in-depth processing of a triplet of intentionally selected hard-to-photograph originals. They are named by the creation procedure, i.e., daguerreotype, ambrotype, and tintype (DAG, AMB, and TIN for short).

**Figure A1:**
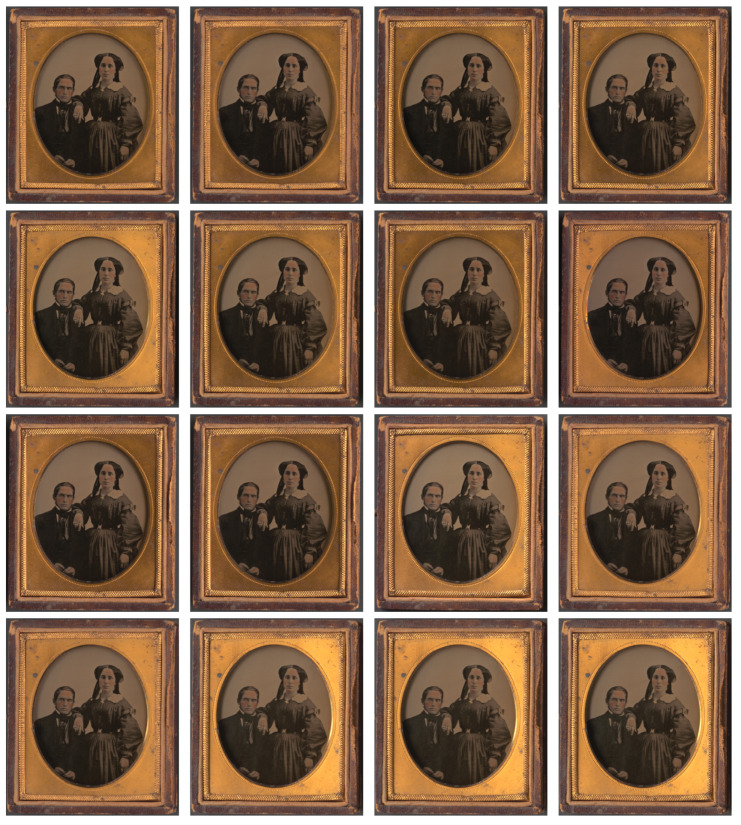
Images of ambrotypes scanned with different light modes. From left to right: *LRFB*, *LR*, *LFB*, *LF*, *LTx*, *LTx 5cm*, *LTx 10cm*, *RTx*, *RTx 5cm*, *RTx 10cm*, *LLa*, *LRFB 15deg*, *LR 15deg*, *LFB 15deg*, *LF 15deg*, *L 15deg*.

Globally, there are a few major initiatives, aggregators, or harvesters of the daily growing volume of online heritage items, including scanned paintings or photographs from more than 7000 heritage institutions. Google Arts and Culture (formerly Google Art Project) [2] is an online platform of high-resolution images and videos of artworks and cultural artifacts from 2000+ partner cultural organizations throughout the world. Europeana [3] is a virtual repository of artworks, literature, cultural objects, relics, and musical recordings/writings from over 2000 European institutions. The Digital Museum Canada (formerly Virtual Museum of Canada) [4] is a virtual collection presenting content from more than 3000 Canadian heritage institutions. Wikipedia GLAM [5] (“galleries, libraries, archives, and museums”, also including botanic and zoological gardens) helps cultural institutions share their resources with the world through collaborative projects with experienced Wikipedia editors, as report the authors of Wikipedia article on Google Arts and Culture. Rare originals from a local collection at the Academy of Fine Arts and Design (AFAD) (Figure A1) will be available through both Europeana and Google Arts and Culture. This will happen after being included in Slovakiana [6], which is guided only in the Slovak language. All three are used for the education of restoration experts, supported by, e.g., a textbook [7]. In the area of digitized old photographing techniques, there is major computer research being done within generative adversarial network (GAN) methods for the digital restoration of damaged photographs, e.g., [8]; in our work, we will not reflect on digital restoration of the photographs.

## 2. Materials and Methods

### 2.1. Materials, a Triplet of Cultural Objects

In total, we studied 3 material originals (cultural objects) and utilized 2 sensors (a scanner and an eye tracker). The primary data in the real-world documented three discoveries in the evolution of photography, daguerreotype, ambrotype, and tintype. Using 16 scanning modes with the given parameters, we obtained secondary data, which are described in the next section and were deposited in a publicly available website under the Creative Commons license [9]. Moreover, the samples of processed images were provided in the same way. Unlike tintypes, daguerreotypes and ambrotypes [10,11] belong to the group of cased photographic objects [12,13,14]. The examined photographs are from the Photographic reference collection at AFAD. The Laboratory of Restoration of Photography deals with research, identification, and the actual restoration of various types of photographic techniques [15]. Emphasis is placed on the gradual acquisition of current non-destructive research techniques and the subsequent choice of restoration intervention. The advantages that we see in using the CRUSE scanning modes versus high-definition photographic images on photographic objects are mainly in the ability to scan objects in very high magnification.

#### 2.1.1. DAG

**Figure A2:**
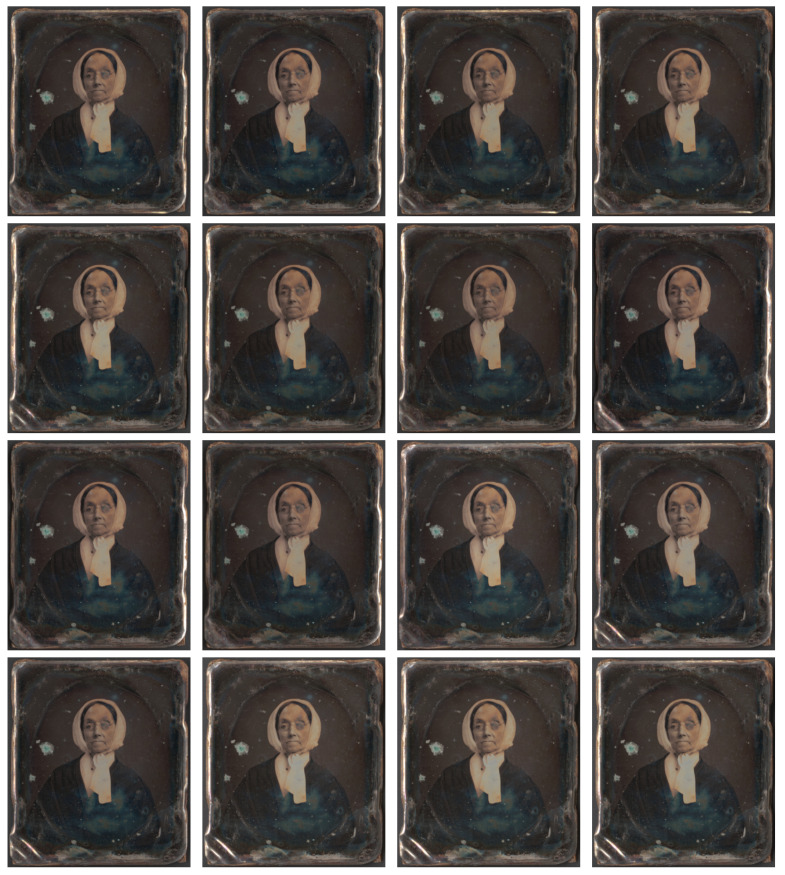
Images of daguerreotypes scanned with different light modes. From left to right: *LRFB*, *LR*, *LFB*, *LF*, *LTx*, *LTx 5cm*, *LTx 10cm*, *RTx*, *RTx 5cm*, *RTx 10cm*, *LLa*, *LRFB 15deg*, *LR 15deg*, *LFB 15deg*, *LF 15deg*, *L 15deg*.

All DAG scans are shown in Appendix A Figure A2. The daguerreotype was the first commercially patented photographic process in 1839 by Louis Daguerre in France. From the material point of view, a daguerreotype is a very complex object consisting of a thin sheet of copper, coated with highly polished silver, and sensitized by iodine fumes exposed in the camera. Afterward, the exposure image is developed in the fumes of mercury and fixed with sodium thiosulfate. As a finish, the object was housed in a protective case, due to its very sensitive nature.

The daguerreotype in Figure A2 was chosen for the examination because it is a good example of this technique. It is a bare plate (missing the protective case), degraded physically and chemically. In general, daguerreotypes have very reflective surfaces, such as a mirror; therefore, it is complicated to photograph them. The scanner has the advantage of minimizing the mirroring effect and the ability to emphasize the actual image.

The following DAG metadata will serve for image documentation, cataloging, and/or retrieval.

Category: historic photo.
Subcategory: reference object for study purposes.
Administered by: University of Fine Arts in Bratislava, Department of Restoration, and Department of Photography and New Media.
Description of the object: The daguerreotype shows a portrait of an elderly woman with glasses and a scarf. The original decorative protective packaging of the daguerreotype is absent.
Technique: daguerreotype
Physical description: number 1, width 70.0 mm, height 80.0 mm.
Degree of authenticity: original.
Legal Status: free.
Inventory number: F-00046-DAG.
Identifier: this field will be supplied according to the future virtual exhibition (e.g., in Europeana).

The original daguerreotype was scanned in *LRFB* (scanning mode first) at a 600 ppi resolution, 48-bit RGB, 0.25 cm was the original height, with 1.10 gain settings. The resulting TIFF file size is about 30 MB and, for further processing, the master scan was converted using GIMP into the PNG file of 21.5 MB. Scan number 1831 refers to the number of scans after the previous scanner calibration. All scans were of the same size, as given by the original width and height, 70 × 80 mm. The naming convention stores the settings, e.g., *Dagero 600.00 ppi LRFB Gain 1.10 0.250 cm 1 831.tif*, see Figure A2 in Appendix A.

#### 2.1.2. AMB

The ambrotype process was invented by Frederick Scott Archer in the 1840s in England. Collodion is poured on a clean sheet of glass, and while still wet, it is dipped into the solution of silver nitrate, exposed in the camera, and fixed. The result is a negative image on the glass. In 1850, Louise Désire Blanquart-Evrard discovered that this image could appear positive when placed on a dark backing. This was possibly due to very little or no silver in the shadows allowing the black background to show through the glass. Due to the very fragile nature of glass, the ambrotypes were housed in protective cases and frames same as daguerreotypes.

The ambrotype in Appendix A Figure A1 is a representative example of the ambrotype image. Ambrotypes are hard to be photographed because they are 3D objects; there is a glass with an image on its verso and the black backing is underneath it. Thus, one looks through the thick glass to see the image underlaid with a black backing (which is not always in direct contact with the image; sometimes it is heavily degraded or is missing completely). All AMB scans are shown in Appendix A Figure A1.

The following AMB metadata will serve for image documentation, cataloging, and/or retrieval.

Category: historic photo.
Subcategory: reference object for study purposes.
Administered by: University of Fine Arts in Bratislava, Department of Restoration, and Department of Photography and New Media.
Description of the object: The ambrotype depicts a portrait of a seated man and a standing woman. The work is heavily hand-colored in the area of the face, hands, and jewelry of the figures. The ambrotype is inserted into a decorative protective cover/cassette, the front closing part of which is absent.
Technique: ambrotype.
Physical description: number 1, ambrotype width with cassette 83.0 mm, ambrotype height with cassette 96.0 mm.
Degree of authenticity: original.
Legal Status: free.
Inventory number: F-00047-AMB.
Identifier: this field will be supplied according to the future virtual exhibition (e.g., in Europeana).

#### 2.1.3. TIN

The ferrotype (tintype) technique belongs to the group of melanotypes and it is inspired by the ambrotype–collodion negative with a black background. Instead of glass, which is overlaid with black paper, velvet, varnish, or paint on a collodion negative background, was designed in 1853 by the French teacher Adolphe Alexandre Martin (1824–1896) to apply the emulsion to a thin black lacquered sheet of iron plate. Black paint was applied to the plate colored paint, varnish (japaning), or enamel. The commonly used term tintype is garbled by transference of meaning because in fact tin was not used, but an iron plate (lat. ferrum). A wet collodion process was used on black iron sheets. Ferrotypes have little contrast, whites are a soft gray, and detail is lost in the shadows. Ferrotype (tintype) was used from 1853 until 1950 when it was still used by street photographers in Turkey or India. As a customer, you could buy a photograph right on the street, made in two minutes.

The following tin metadata will serve for image documentation, cataloging, and/ or retrieval.

Category: historic photo.
Subcategory: reference object for study purposes.
Administered by: University of Fine Arts in Bratislava, Department of Restoration, and Department of Photography and New Media.
Description of the object: The ferrotype shows a portrait of a seated man and a standing woman. The work is in the area the faces and hands of the characters are gently colored by hand.
Technique: ferrotype, tintype.
Physical description: number 1, width 61.0 mm, height 103.0 mm.
Degree of authenticity: original.
Legal Status: free.
Inventory number: F-00048-TIN.
Identifier: this field will be supplied according to the future virtual exhibition (e.g., in Europeana).

**Figure A3:**
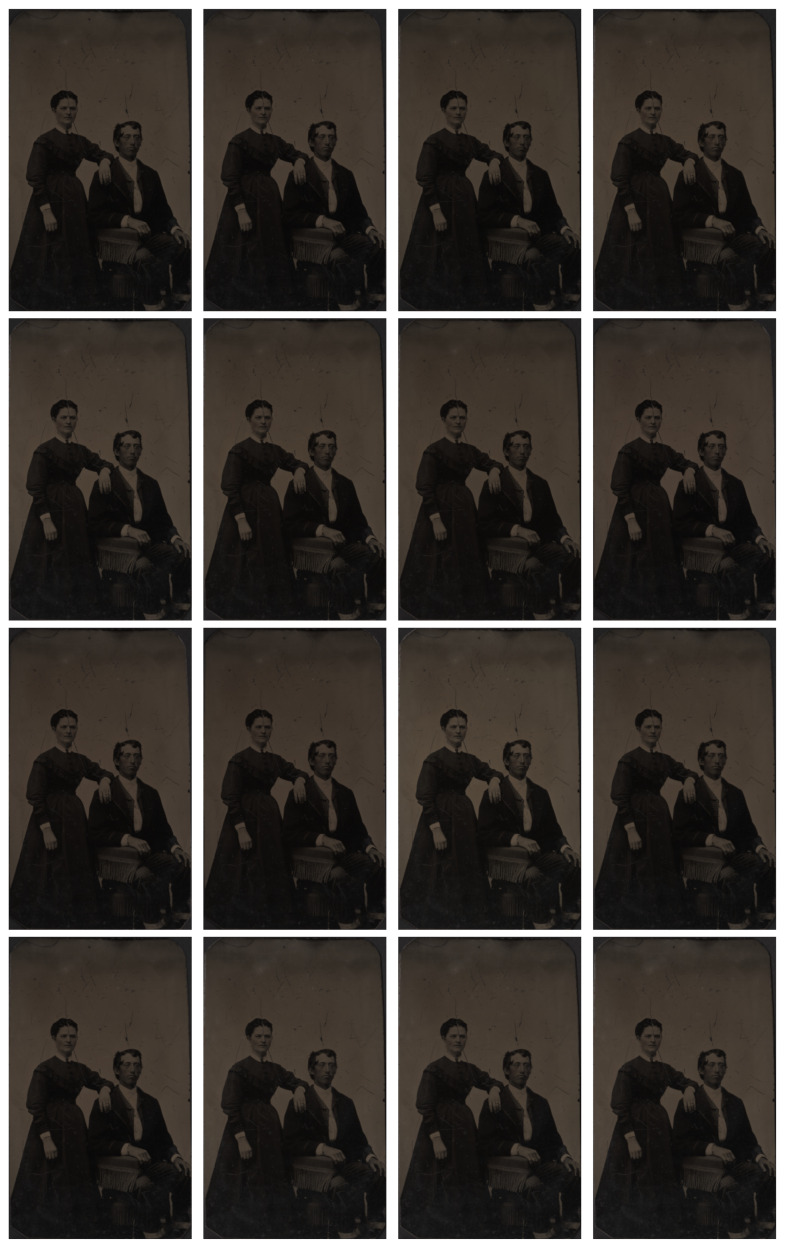
Images of tintypes scanned with different light modes. From left to right: *LRFB*, *LR*, *LFB*, *LF*, *LTx*, *LTx 5cm*, *LTx 10cm*, *RTx*, *RTx 5cm*, *RTx 10cm*, *LLa*, *LRFB 15deg*, *LR 15deg*, *LFB 15deg*, *LF 15deg*, *L 15deg*.

All TIN scans are shown in Appendix A Figure A3.

### 2.2. Methods, Modeling the Scanner Focusing, InfoVis, Eye Tracking

We divide the originally designed workflow into naturally given groups of methods. 1. We explain the standard scanner settings of a given push-broom camera [16] and the mathematical model of the manual sharpening process, based on the Fourier transform. 2. Within a data state reference model in information visualization [17], we visualize and distinguish properties of scanning modes. This was done by transforming the image differencing problem to a well-studied assignment problem, using Harris corners [18] or Co-SIFT feature points [19] 3. We analyze the differences between various scans by multiple image quality metrics. 4. The qualitative evaluation by restoration experts from the Laboratory of Restoration of Photography [15] is supported by eye-tracking measurements of their perception.

#### 2.2.1. Contactless Scanning

The CRUSE scanner CS 220ST1100 (CCD “14.400 Pixel Tri Linear RGB Line-Sensor” with Schneider optics) offers 4 light sources *LRFB* (left, right, front, back) and *LRFB* scanning mode is the default one for heating the lamps, which requires 30 min. This starting mode is the fastest one. Another mode, recommended for scanning artwork, is *LTx*, which better documents the fine relief of the given original. It is possible to scan the given scan-line of real artwork under constant illumination accurately up to 1200 ppi (pixels per inch) in the TIFF format. The scanned original weight may be up to 300 kg, measuring 120 × 180 × 30 cm [20]. The current software CSx does not create a 3D model, but allows for precise scanning with variable illumination, 15° sensor rotation, and variable depth of field, thus offering dozens of scan versions of a single original. The ultimate and unique truth does not exist here. Consequently, there are many best scans, each one for a given mode and settings. The wooden decors illustrate a sample from natural heritage [21]. From cultural heritage we selected hard-to-distinguish cases, such as ambrotypes, daguerreotypes [19,21], synagogue plans, or handmade papers [22]. For educational purposes, Comenius University students created virtual herbarium, 3D models using photogrammetry, depth of field, or shadows [23].

The CRUSE scanner offers different light modes: *LRFB*, *LR*, *LFB*, *LF*, *LTx*, *LTx 5cm*, *LTx 10cm*, *RTx*, *RTx 5cm*, *RTx 10cm*, *LLa*, *LRFB 15deg*, *LR 15deg*, *LFB 15deg*, *LF 15deg*, *L 15deg*. They can be subdivided into several groups using several criteria, i.e., symmetric, rotated, and shifted. Their effect is captured in scans. Given pairs of scans are hard to distinguish by the naked eye. Using computer vision methods we can either segment the objects to support the semantics or mimic the human vision by low-level operations, adding points and edges first or image evaluation then. The computed and perceived versions of interpretation can be consequently compared to gain more insight. We elaborate on this approach in more detail in the following parts.

#### 2.2.2. Perceptually Meaningful Dot Patterns and Density

The motivation for adding edges into the image data after Toussaint in 1980 [24]:

In… computational approaches to perception… it is desired to find some structure in the form of edges connecting the subset of the pairs of points... such that the final graph obtained is perceptually meaningful in some sense.

The proposed solution by Toussaint was a relative neighborhood graph (RNG), a superset of the minimum spanning tree (MST), and the subset of Delaunay triangulation (DT). Our idea was to relax from the connected visual descriptor or spanner (MST, RNG) by connecting the dots with the “Hungarian” edges [25,26]. For adding dots, we experimented with searching the gray level Harris corners [18] and concurrent color channels coSIFT [27]. The latter performed better on Cruse scans and already the density of Harris or coSIFT corners indicated “interesting parts” in scanned wooden decors. For more details on the Hungarian edges approach, refer to [19,21]. Methodologically, adding corners and edges can serve for image (or scanning mode) differentiation in terms of the data state reference model in information visualization [17]. The primary scan is analytically abstracted with added feature points, which are consequently visualized by adding edges and displayed. Note that DT edges can be too long for certain dot configurations but the Hungarian edges’ total length is warranted to be minimal.

Technically, three sequential processes occur with visual data: acquisition, representation, and processing/simulation of human vision. These processes support understanding and ultimately lead to a decision. Metaphorically, an inexperienced cultural object owner might view the resulting versions in a similar way to how people used to observe the night sky, i.e., by identifying the brightest stars in a given area, connecting them with edges, and then searching for suitable symbolism. Our approach can be helpful in the early stages by providing different corners and edges.

In this study, we move away from focusing on edges because we can directly associate the density of dots with appealing image components, such as salient regions, or semantic primitives, such as human faces. These elements can ultimately contribute to the overall meaning of a portrait.

Image quality measuring is based on an assumption that there exists one ideal, ground truth image. If such an image is not available, we have to give up the algorithmic problem-solving, inevitably opt for the heuristic approach, and select one of the available options as a relatively quality prototype. For multiple sensors, the quality evaluation of nearly identical images was proposed, e.g., in [28]. This scenario can be observed in CRUSE scanning multiple modes offered by the CSx software tool. The authors in [21] compared *LRFB* scans (in the role of ground truth) against other ones, using the Harris corner detector, Hungarian edges, and SSIM (structural similarity index measure) comparisons. This detector suffered from a low count of Harris corners for certain hard-to-scan images. Our goal in coSIFT [27] research was to improve the gray level method [21] by a more color-sensitive corner detector, which we describe in detail below. These data will be used to compute the coSITF heatmaps, which will be confronted with the measured heatmaps from the evaluation of eye-tracking sessions.

#### 2.2.3. Eye Tracking Session

To find out which parts of the scanned images are important or interesting for the experts from the Academy of Fine Arts and Design, we recorded their eye movements during an eye-tracking session. The data were collected during a free-viewing of three different digitized photographs, scanned with different scanning modes.

**Figure 1:**
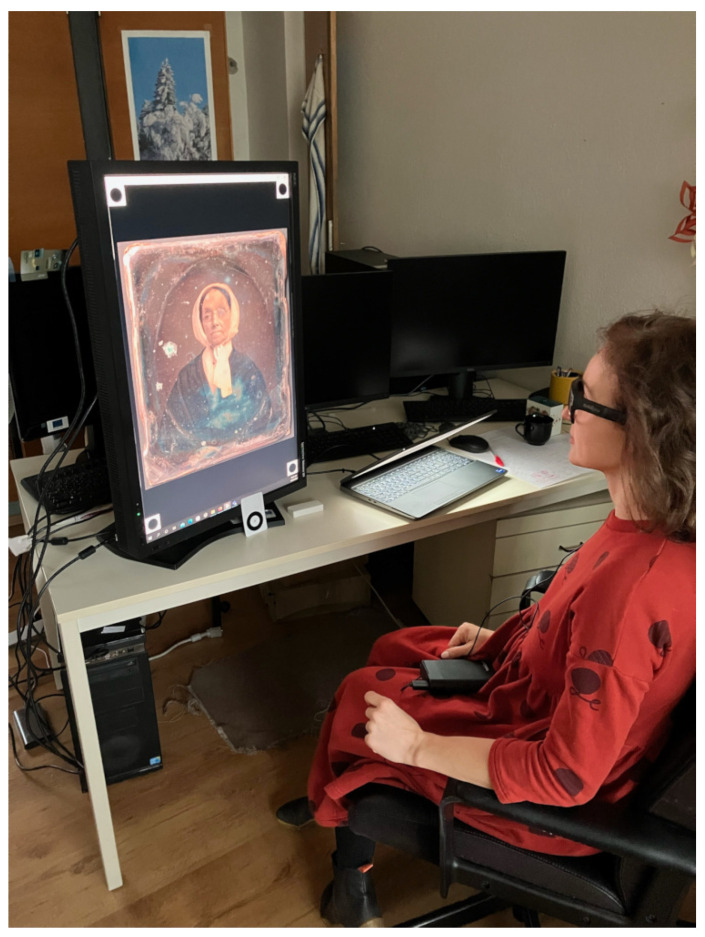
Image of the eye-tracking session at Comenius University. The participant, Zuzana Berger Haladová agrees with the publishing of her photo here. Photo by Zuzana Černeková, 2022.

Two restoration professionals with at least five years of experience in the field participated in the study. The participants were seated in front of a computer monitor and asked to view scans of Ambrotype, Daguerreotype, and Tintype photographs. The eye-tracking equipment recorded the participants’ gaze patterns as they viewed the scans. The recording took 5 minutes for each participant. The eye-tracking data were analyzed to determine the visual attention patterns of the participants. The setup of the session can be seen in Figure 1.

**Figure 2:**
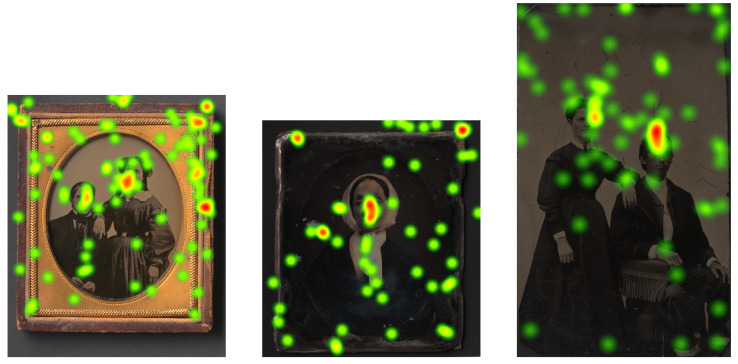
These three heatmaps show looking behavior of two restoration expert participants to the Ambrotype, Daguerreotype and Tintype scanned with the *LLa* scanning mode.

At the beginning of each recording, the eye tracker was calibrated. The calibration precision for the first participant was 92% for the second one 95%. The focus of the visual attention of participants can be visualized using heatmaps. They show how looking is distributed over the images. heatmaps obtained from our eye tracking session can be seen in Figure 2. The heatmaps were generated using the software Tobii Pro Lab [29].

#### 2.2.4. Eye Tracker Tobii

**Figure 3:**
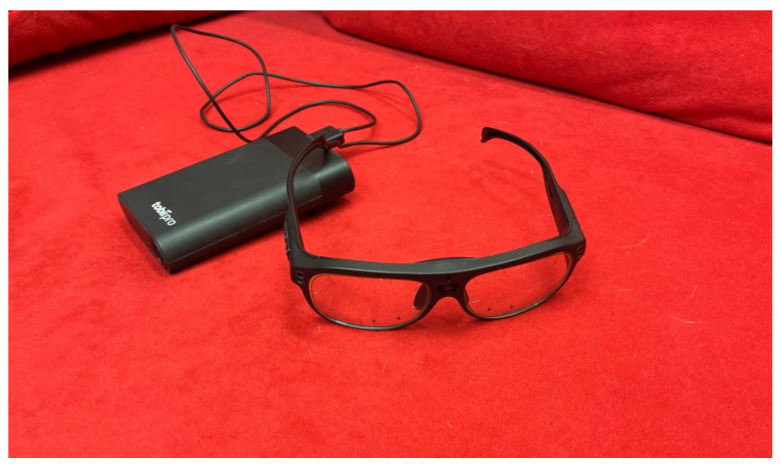
Tobii Pro Glasses 3.

We used Tobii Pro Glasses 3 (Figure 3), the third-generation wearable eye-tracking glasses, to capture areas of interest in the images. Tobii Pro 3 glasses provide robust eye tracking and accurate gaze data while giving users freedom of movement. The glasses contain 16 illuminators and 4 eye cameras integrated into scratch-resistant lenses, to provide optimal eye-tracking performance and create an unobstructed view for the user. The scene camera offers a wide field of view (106° H: 95°, V: 63°).

### 2.3. Co-SIFT

**Figure 4:**
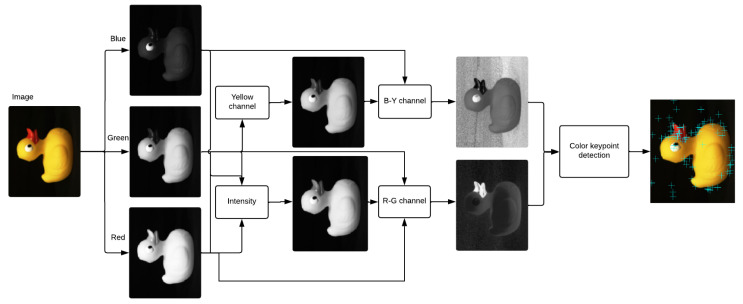
The pipeline of the Co-SIFT [27].

Although the color is perceived as an irreplaceable element describing the world around us, the techniques for extracting the local features are mostly based on the description of the shape, while the color information is entirely ignored. The pipeline of the proposed Co-SIFT [27] algorithm is illustrated in Figure 4. The key idea of our solution is to incorporate color information from the image into the SIFT method by replacing grayscale information so that key points are detected on two separate chromatic channels separately (red–green (RG) and a yellow–blue (YB)) and the achromatic brightness channel. Individual steps of the algorithm are described in detail in the following subsections.

#### 2.3.1. Pre-Processing

Stephen Engel et al. in [30] examined the human visual cortex and its color matching using magnetic resonance. The human visual cortex processes signals from photoreceptors on the retina of the eye and interprets the color information. The authors [31] experimentally found that the strongest response is to red–green stimuli. In yellow-blue stimuli, the reaction is also strong, but compared to red–green stimuli, it decreases rapidly. The combination of the trichromatic process and the opponent color-coding process was until recently considered impossible. The trichromatic process, however, speaks of composing colors from several cones, the process of the opponent’s colors, on the other hand, involves finding the colors from their differences. However, the eye works on a much more complicated level, and these two processes perfectly combine. Therefore, as a basic model for our method, we chose, an approach based on experiments in [30], using chromatic opponent channels proposed in [32] and trichromatic color theory, for color image processing and the SIFT method.

The intensity channel (1) is computed as the weighted average of the *R*, *G*, and *B* values, where the weights were acquired by measuring the intensity perceived by the people with undisturbed trichromatic vision. The weights are the same as used in the standard sRGB space by [33]

(1) I=0.2126·R+0.7152·G+0.0722·B.

In this color space, the two chromatic channels (RG and YB) proposed in [34] are normalized by the intensity channel, which removes the effect of intensity variations. The channels are defined as follows

$$RG=\frac{\left(R-G\right)}{I}$$

and

$$YB=\frac{\left(B-Y\right)}{I},$$

where

$$Y=\frac{\left(R+G\right)}{2}.$$

Now we proceed with the detection of the interesting points in the chromatic channels.

#### 2.3.2. Interesting Point Detection

SIFT algorithm introduced by [35] consists of a scale and rotation invariant detector and a HOG (histogram of oriented gradients) descriptor. SIFT detector uses a Gaussian scale pyramid. The image is scaled to *K* sizes–octaves. Each octave is then recurrently filtered by a 2D Gaussian.

Two consecutive images in each octave are then subtracted and the resulting N−1 DoG (difference of Gaussian) images are approximations of LoG (Laplacian of Gaussian) images. The points of interest are identified in the 3 × 3 × 3 neighborhood in the DoG space. The octave in which the interesting point was found represents the “scale” of the IP and determines the size of the neighborhood for descriptor extraction of that point.

In our method, the Co-SIFT interesting point detection is applied directly to each opponent’s chromatic channel.

**Figure 5:**
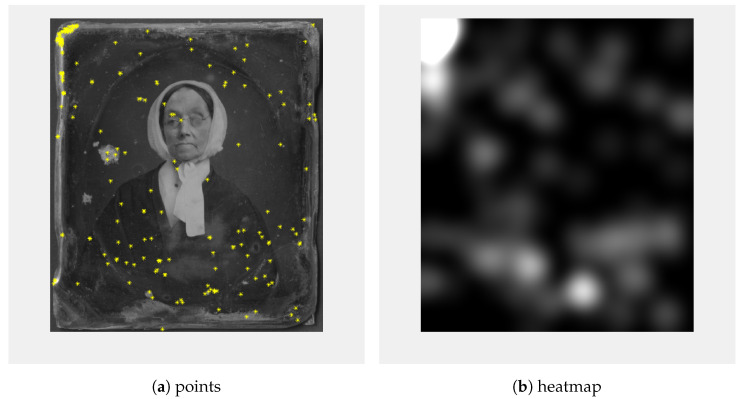
A total 200 points (**a**) and heatmap (**b**) calculated using the Harris corner detector.

**Figure 6:**
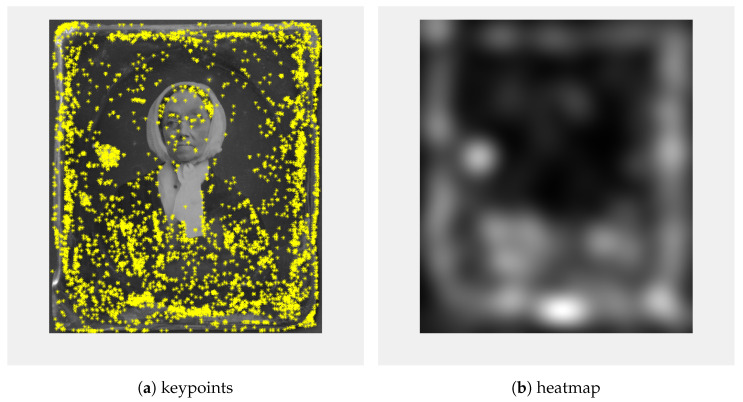
Keypoints (**a**) calculated using Co-SIFT detector, heatmap (**b**) calculated from the Co-SIFT keypoints.

As feature detectors, we used co-SIFT and Harris corner detectors [18]; 200 Harris corners were calculated (Figure 5a) and a heatmap (Figure 5b) was created so that for every point a circle blurred with Gaussian blur was added. Similar to the keypoints calculated using Co-SIFT (Figure 6a), the heatmap (Figure 6b) was created.

The number of co-SIFT features, resp. keypoints for input images varied from 4 to 5143.

## 3. Manual Focus of the Push Broom-like Scanners

Scanners with lenses that capture images in a similar way to cameras do not always have autofocus capability. If we want the resulting scanned image to be sharp enough, we need to know the height of the object to be scanned and make sure that the optical system is well-adjusted and calibrated. Since it is not always easy to meet both conditions, scanners often provide a manual focusing option that uses one of the image sharpness functions mentioned later in this paper.

The sharpness function calculates the sharpness measure or degree of sharpness using the pixel values of the captured image. The value of the sharpness function varies depending on the distance of the lens from the image sensor. The distance of the lens from the sensor is provided by a precision stepper motor, which searches for the position at which the image captured by the sensor is the sharpest. A suitable sharpness function should meet at least the following criteria [36]:

- Unimodality—the sharpness function contains one significant extreme;
- Accuracy—the maximal sharpness is at the extreme of the function;
- Monotonicity—the sharpness function is monotonic at a sufficient distance from the extreme.

There are many sharpness functions. According to [37,38], they can be divided into functions using differences, image convolution, Fourier transform, image entropy, and others. All sharpness functions exploit the fact that details in an image are more visible when the image is sharper.

When looking for an extreme, due to oscillations (rapid changes) of the sharpness function, it is possible to use smoothing of the function, e.g., using a moving average [39]. Since a color image usually consists of three color components *R*, *G*, *B*, it is necessary to either convert the image to grayscale or calculate the sharpness function for only one color component [40].

In this section, we will consider the image of size *M* × *N* as a 2D function f(x,y), for x=0,1,…,M−1 and y=0,1,…,N−1. The x,y values are the spatial coordinates and the value of f(x,y) at any point (x,y) denotes the intensity at that point (pixel).

### 3.1. Sharpness Functions Based on Differences

These functions compute a measure of sharpness using the sum of the differences of the values of adjacent pixels in either the horizontal or vertical direction, or in both directions simultaneously [36,37,38,40]

$$\begin{array}{cc} S_{df,1}\left(x,y\right) & =\sum_{x=0}^{M-k-1}\sum_{y=0}^{N-1}\left|I(x+k,y)-I(x,y)\right|\;\mathrm{or}\\ S_{df,1}\left(x,y\right) & =\sum_{x=0}^{M-1}\sum_{y=0}^{N-k-1}\left|I(x,y+k)-I(x,y)\right|,\\ S_{df,2}\left(x,y\right) & =\sum_{x=0}^{M-k-1}\sum_{y=0}^{N-k-1}\left(\left|I(x+k,y)-I(x,y)\right|+\left|I(x,y+k)-I(x,y)\right|\right). \end{array}$$

In general, the difference between more than two rows or columns can also be used, which partially eliminates the sensitivity of these measures to noise in the image [36]. The value of *k* is usually chosen as 1 or 2, but [36] states that a higher value can be used.

### 3.2. Sharpness Functions Based on Image Convolution

These sharpness functions use image filters that highlight edges in the image at an appropriately chosen threshold *T* [36,37,38,39,40].

The resulting sharpness function is computed using the convolution operator “∗” of the image function I(x,y) with the given convolution function ω(x,y)

(2) $$I^{\prime }\left(x,y\right)=\left(\omega *I\right)\left(x,y\right)=\sum_{r=-k}^{k}\sum_{s=-k}^{k}\omega (r,s)I(x-r,y-s),\;\mathrm{for}\;k=1,2,...$$

Of the relatively large number of sharpness functions using convolution, the following are the most used:

$$\begin{array}{cc} S_{ic,1}\left(x,y\right) & =\sum_{x=1}^{M-2}\sum_{y=1}^{N-2}\left|L(x,y)\right|,\;\mathrm{for}\;\left|L(x,y)\right|>T,\\ S_{ic,2}\left(x,y\right) & =\sum_{x=1}^{M-2}\sum_{y=1}^{N-2}\left(G_{x}^{2}\left(x,y\right)+G_{y}^{2}\left(x,y\right)\right),\;\mathrm{for}\;G_{x}^{2}\left(x,y\right)+G_{y}^{2}\left(x,y\right)>T^{2}, \end{array}$$

where $L\left(x,y\right)=\left(L*I\right)\left(x,y\right),\;L=\left(\begin{array}{ccc} 0 & -1 & 0\\ -1 & 4 & -1\\ 0 & -1 & 0 \end{array}\right)$,

$G_{x}\left(x,y\right)=\left(S_{x}*I\right)\left(x,y\right),\;S_{x}=\left(\begin{array}{ccc} -1 & 0 & 1\\ -2 & 0 & 2\\ -1 & 0 & 1 \end{array}\right)$ and

$G_{y}\left(x,y\right)=\left(S_{y}*I\right)\left(x,y\right),\;S_{y}=\left(\begin{array}{ccc} -1 & -2 & -1\\ 0 & 0 & 0\\ 1 & 2 & 1 \end{array}\right).$

### 3.3. Sharpness Functions Based on Fourier Transform

Sharpness functions using the Fourier transform are computationally intensive but are sufficiently robust to the moderate noise in the image coming from image sensors. These functions require that the image be first transformed into the frequency domain using a discrete Fourier transform (DFT) [36,38,40,41] or a discrete cosine transform (DCT) [42]. In a sharp image, the higher frequency values are larger, in an unfocused image they are smaller. Similarly, the values at lower frequencies are larger for an unfocused image and smaller for a focused image [41]. To define sharpness functions, it is often convenient to use only a certain frequency interval $\left[T_{low},T_{high}\right]$ [36].

The discrete Fourier transform F(u,v) of the image function I(x,y) is expressed using the formula

$$F\left(u,v\right)=\frac{1}{MN}\sum_{x=0}^{M-1}\sum_{y=0}^{N-1}I\left(x,y\right)e^{-i2\pi \left(u\frac{x}{M}+v\frac{y}{N}\right)},$$

but to calculate the sharpness function we use a formula in which the DFT is written using the real and imaginary components

F(u,v)=1MN∑x=0M−1∑y=0N−1I(x,y)cos2πuxM+vyN=−i1MN∑x=0M−1∑y=0N−1I(x,y)sin2πuxM+vyN=Re(F(u,v))−iIm(F(u,v)).

Based on the previous formula, we calculate the sharpness function using one of the following formulas

$$\begin{array}{cc} S_{ft,1}\left(x,y\right) & =\sum_{u=0}^{M-1}\sum_{v=0}^{N-1}\left|F(u,v)\right|,\;\mathrm{while}\;T_{low}<u+v<T_{high},\\ S_{ft,2}\left(x,y\right) & =\sum_{u=0}^{M-1}\sum_{v=0}^{N-1}\left|\left|F(u,v)\right|\cdot \arg\left(F\left(u,v\right)\right)\right|, \end{array}$$

where F(u,v)=Re2(F(u,v))+Im2(F(u,v)), arg(F(u,v))=arctanIm(F(u,v))Re(F(u,v)).

### 3.4. Experiments with Manual Focusing

For use in photography, sharpness measures based on differences and measures based on image convolution are the most suitable, according to [37,38]. Sharpness functions based on the Fourier transform are sufficiently robust not only to the surface texture of the captured object and different lighting conditions but also to noise in the image [41,42]. However, their accuracy is slightly worse than that of the previously mentioned methods.

**Figure 7:**
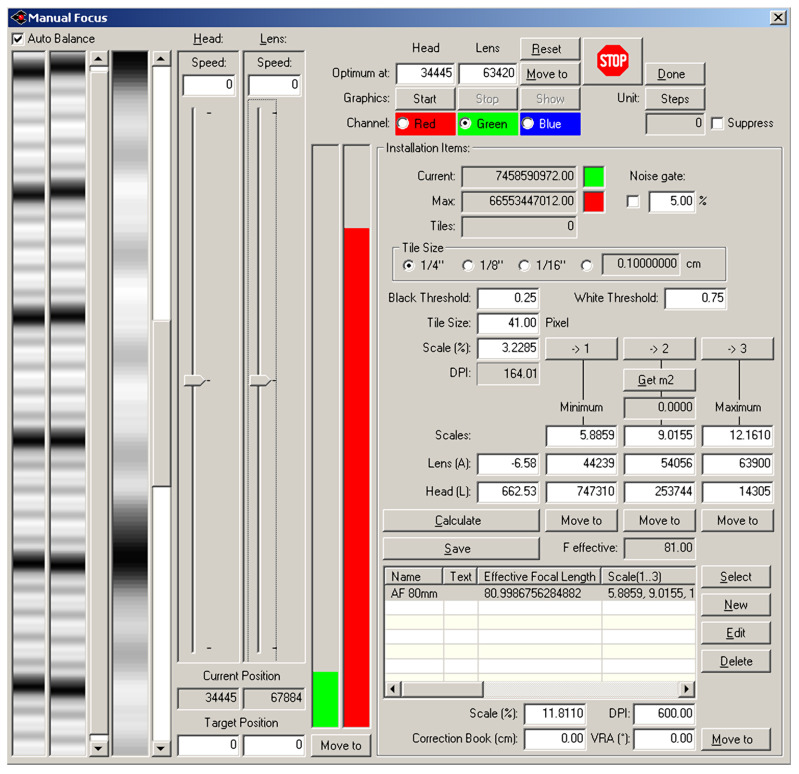
Cruse manual focus dialog for the unfocused picture.

**Figure 8:**
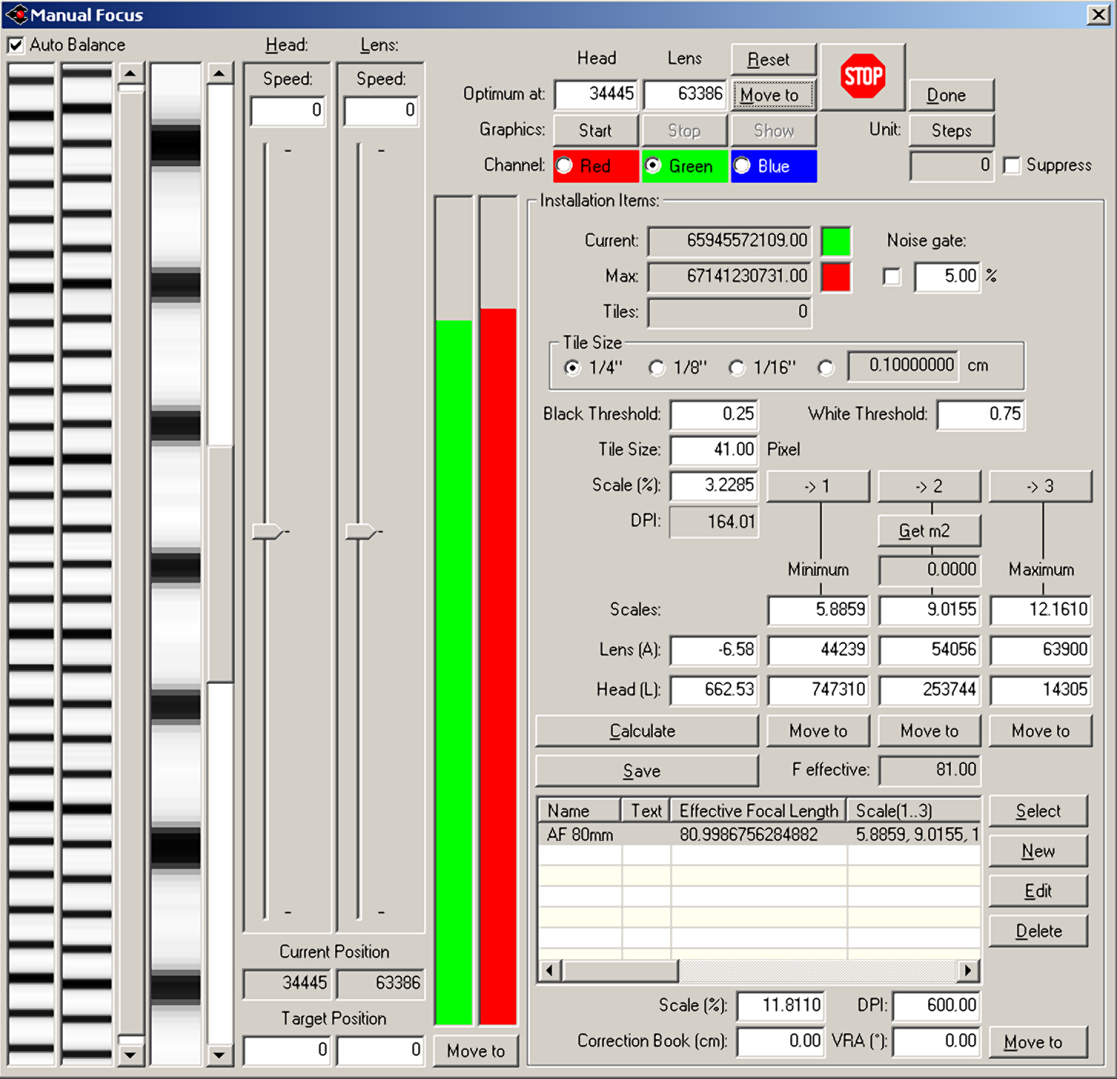
Cruse manual focus dialog for the focused picture.

**Figure 9:**
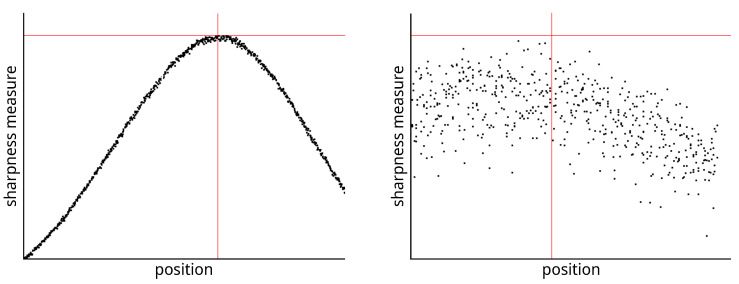
Cruse Focus Value Diagram: graph of sharpness function (**left**), detail of the graph with noise (**right**).

The Cruse scanner uses one of the above-mentioned sharpness functions for manual focusing. It has an advanced interface for manual focusing, which can be seen in Figure 7 and Figure 8. In this interface, it is possible to change the distance of the lens from the sensor (current position) by adjusting the speed of the stepper motor (lens speed) that moves the lens or the entire sensor head (head speed). We selected the green color channel to calculate the sharpness function. We set the minimum threshold (black threshold) to 0.25 and the maximum (white threshold) to 0.75. The scanner control algorithm then calculated the sharpness measure (installation items—current) for the current moving lens position and continuously displayed the calculated maximum value (installation items—max). After verifying the maximum value using the graph of sharpness function (see Figure 9), we set the scanning head and lens to the position (Optimum At:) where the sharpness function had the largest value.

A magnified picture of one column of the unfocused scanned image can be seen in Figure 7, and a picture of the focused image can be seen in Figure 8. The sharpened image has larger sharpness values, which are manifested by a distinct and clearly delineated alternation of dark and light bands.

**Figure 10:**
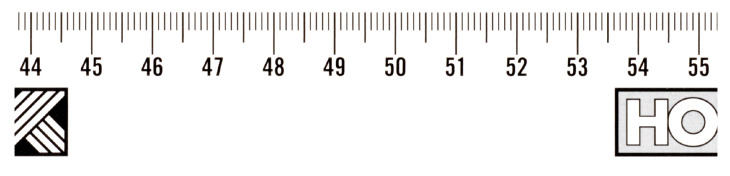
Picture of scanned black-and-white ruler.

**Figure 11:**
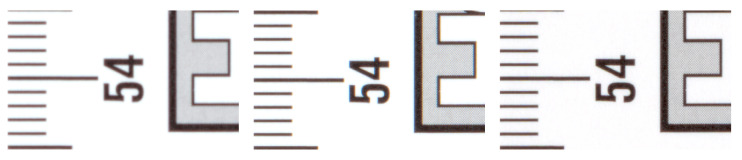
Unfocused with h=0.0 cm (**left**), unfocused with h=1.4 cm (**center**), focused with h=0.0 cm (**right**).

We verified the manual focusing procedure by scanning on an inaccurately calibrated scanner on multiple objects and at different resolutions. One of them was a black and white ruler (see Figure 10) with height h=0.0 cm, for which we used a scanner resolution of 600 ppi. The 4× magnified cutout (see Figure 11 left) shows a poorly focused image. The scanner makes it possible to obtain a sharper image by setting a different height than the actual height of the object being scanned, but at the cost of increased chromatic aberration (see Figure 11 in the middle). After manual sharpening using the sharpness function $S_{ft,1}\left(x,y\right)$, we obtained a significantly sharper image without chromatic aberration (see Figure 11 right).

The CRUSE Synchron Table scanner makes it possible to manually focus the image even in the case of insufficient calibration or incorrectly estimated height of the scanned object with complex relief. For this purpose, it uses a fairly robust sharpness function based on the aforementioned list of sharpness functions.

## 4. Comparison of Images Captured in Different Scan Modes of the CRUSE Synchron Table Scanner

As we mentioned earlier, the CRUSE Synchron Table scanner has many lighting modes to choose from. Our goal was to compare different lighting modes and select one that captures the small surface damage of the scanned objects sufficiently well. Thanks to its design, the scanner allows us to scan objects that are not completely flat with sufficient sharpness. This is made possible because when scanning with a resolution of 600 ppi, the depth of field is around 1.0 cm, and at a lower resolution, it is even slightly larger.

Several methods can be used to compare the similarity of the two images. The simplest of them, such as MSE, RMSE, and similar ones, use well-known statistical measures such as the mean squared error, mean absolute error, mean relative error, and the standard deviation or correlation. We could not use simple statistical measures when comparing images, since different lighting modes produce images not only with different average pixel intensities but sometimes with different colors as well. This is most evident when scanning glossy surfaces. Useful measures could be UIQI (universal image quality index), SSIM (structural similarity index measure) and its variations, image entropy value, or histogram comparison, as shown by other authors when comparing two images [43]. It is also possible to use other, less well-known, measures based on spectral distance or the human visual system (HVS) [44]. In our comparison, we did not have to take into account measures that compare images of different sizes or images that are scanned with a different rotation.

Before comparison, we converted all evaluated images into 8-bit grayscale images with sRGB color profile. To convert from a color image to a grayscale image, we used the Equation (1) that takes into account the human perception of color intensity [45].

In the following text, we compare two equal-sized grayscale images I(x,y) and J(x,y) of size M×N, with pixel intensities from 0 to 255, for x=0,1,2,…,M−1 and y=0,1,2,…,N−1.

The simple differences in the image pixels generally do not give good results, so we used other measures to compare the two images.

### 4.1. Universal Image Quality Index

The first measure we used to compare two images was the Wang and Bovik UIQI measure [46], which uses common image measures, such as mean and standard deviation. This measure simultaneously compares the structural distortion (correlation loss)

$$s\left(I,J\right)=\frac{\sigma_{IJ}}{\sigma_{I}\sigma_{J}},$$

luminance distortion

$$l\left(I,J\right)=\frac{2\mu_{I}\mu_{J}}{\mu_{I}^{2}+\mu_{J}^{2}},$$

and contrast distortion

$$c\left(I,J\right)=\frac{2\sigma_{I}\sigma_{J}}{\sigma_{I}^{2}+\sigma_{J}^{2}}$$

in the resulting formula for calculating the similarity index of the two compared images

(3) $$\mathrm{UIQI}\left(I,J\right)=s\left(I,J\right)\cdot l\left(I,J\right)\cdot c\left(I,J\right)=\frac{4\sigma_{IJ}\mu_{I}\mu_{J}}{\left(\mu_{I}^{2}+\mu_{J}^{2}\right)\left(\sigma_{I}^{2}+\sigma_{J}^{2}\right)},$$

where $\mu_{I}$, $\mu_{J}$ are the average pixel values of the first and second image, $\sigma_{I}$ and $\sigma_{J}$ are standard deviations for the first and second image, and finally $\sigma_{IJ}$ is the covariance of both images.

### 4.2. Structural Similarity Index Measure

The second measure we used was Wang et al. [47,48] SSIM measure, which is derived from UIQI by adding stabilization constants $C_{1},C_{2},C_{3}$ to the structural distortion

$$s\left(I,J\right)=\frac{\sigma_{IJ}+C_{1}}{\sigma_{I}\sigma_{J}+C_{1}},$$

luminance distortion

$$l\left(I,J\right)=\frac{2\mu_{I}\mu_{J}+C_{2}}{\mu_{I}^{2}+\mu_{J}^{2}+C_{2}},$$

and contrast distortion

$$c\left(I,J\right)=\frac{2\sigma_{I}\sigma_{J}+C_{3}}{\sigma_{I}^{2}+\sigma_{J}^{2}+C_{3}},$$

which also serves as a tolerance factor for the comparison.

In the formula for SSIM, the powers of alpha, beta, and gamma for the above functions are also used:

$$\mathrm{SSIM}\left(I,J\right)=l{\left(I,J\right)}^{\alpha }\cdot c{\left(I,J\right)}^{\beta }\cdot s{\left(I,J\right)}^{\gamma }.$$

For easier calculation of the SSIM formula, Wang et al. calculated the last constant as $C_{3}=C_{2}/2$ and they chose α=β=γ=1. Since the constants $C_{1},C_{2}$ depend on the given range *L* of pixel intensities of the compared images, they are calculated from the formula $C_{1}={\left(K_{1}\cdot L\right)}^{2}$, $C_{2}={\left(K_{2}\cdot L\right)}^{2}$, where $K_{1},K_{2}\leq 1$. After setting these constants, we calculate the similarity measure of the two images using the formula:

(4) $$\mathrm{SSIM}\left(I,J\right)=\frac{\left(2\mu_{I}\mu_{J}+C_{1}\right)\left(2\sigma_{IJ}+C_{2}\right)}{\left(\mu_{I}^{2}+\mu_{J}^{2}+C_{1}\right)\left(\sigma_{I}^{2}+\sigma_{J}^{2}+C_{2}\right)}.$$

In practice, it is more convenient to compare two images not globally (all at once), but locally [48], in separate pieces using a suitably chosen sliding window, which gradually passes through all the pixels (x,y) of the compared images *I* and *J*.

To calculate the average intensity $\mu_{I}$ of pixel I(x,y) and $\mu_{J}$ of pixel J(x,y) in a window of size (2k+1)×(2k+1) with weights ω(x,y), we used the formulas

$$\mu_{I}=\sum_{r=-k}^{k}\sum_{s=-k}^{k}\omega (r,s)I(x-r,y-s),\;\mathrm{for}\;k=1,2,...$$

and

$$\mu_{J}=\sum_{r=-k}^{k}\sum_{s=-k}^{k}\omega (r,s)J(x-r,y-s),\;\mathrm{for}\;k=1,2,...$$

For the standard deviation, we used the formulas

$$\sigma_{I}={\left(\sum_{r=-k}^{k}\sum_{s=-k}^{k}\omega \left(r,s\right){\left(I\left(x-r,y-s\right)-\mu_{I}\right)}^{2}\right)}^{1/2}$$

and

$$\sigma_{J}={\left(\sum_{r=-k}^{k}\sum_{s=-k}^{k}\omega \left(r,s\right){\left(J\left(x-r,y-s\right)-\mu_{J}\right)}^{2}\right)}^{1/2}.$$

Finally, for the covariance, we used the formula

$$\sigma_{IJ}=\sum_{r=-k}^{k}\sum_{s=-k}^{k}\omega \left(r,s\right)\left(I\left(x-r,y-s\right)-\mu_{I}\right)\left(J\left(x-r,y-s\right)-\mu_{J}\right).$$

The weights ω(x,y) were calculated using the Gaussian function

$$g_{\sigma }\left(x,y\right)=\frac{1}{2\pi \sigma^{2}}e^{-\frac{{\left(x-\mu_{I}\right)}^{2}+{\left(y-\mu_{J}\right)}^{2}}{2\sigma^{2}}}.$$

The weights chosen in this way ensure that the local values ${\mathrm{SSIM}}_{xy}\left(I\left(x,y\right),J\left(x,y\right)\right)$ of neighboring pixels do not change abruptly.

The resulting SSIM(I,J) measure is then calculated as the standard mean of local ${\mathrm{SSIM}}_{xy}$ values:

(5) $$\mathrm{SSIM}\left(I,J\right)=\frac{1}{MN}\sum_{x=0}^{M-1}\sum_{y=0}^{N-1}{\mathrm{SSIM}}_{xy}\left(I\left(x,y\right),J\left(x,y\right)\right).$$

In our experiments, we used $K_{1}=1.0$, $K_{2}=0.04$, L=255, window size 5×5 and σ=1.0 to calculate the similarity of the two images. Compared to the recommended value of $K_{1}=0.01$, in the work of Wang et al. [47], we chose a much higher value of $K_{1}$ to disregard very small structural changes, such as dust grains or very small scratches, in the comparisons. The smaller window size and smaller σ value (compared to the standard values in Wang et al.) provided finer details of the drawing on the displayed SSIM difference map.

### 4.3. Edge Strength Similarity Image Quality Metrics

The third measure we used to compare images scanned in different light modes was the ESSIM (edge strength similarity image quality metrics) by Zhang et al. [49]. This measure exploits the fact that the most significant features of an image are the boundaries (edges) of objects. The authors used Scharr’s gradient operator (filter), which they modified to detect not only horizontal and vertical edges but also diagonal edges.

Zhang et al. [49] used the directional derivative of $\partial I_{j}\left(x,y\right)$ in pixel (x,y) for direction j=1,2,3,4 and define the edge “strength” in the diagonal up (j=2) and diagonal down directions (j=4):

$$E_{2,4}\left(I\left(x,y\right)\right)={\left|\partial I_{2}\left(x,y\right)-\partial I_{4}{\left(x,y\right)}^{2}\right|}^{p},$$

then the edge strength for the horizontal direction (j=1) and the vertical direction (j=3)

$$E_{1,3}\left(I\left(x,y\right)\right)={\left|\partial I_{1}\left(x,y\right)-\partial I_{3}{\left(x,y\right)}^{2}\right|}^{p},$$

where *p* is a suitable scaling parameter that affects the edge strength.

Since the human visual system is sensitive to the direction of the edge that is more intense, Zhang et al. [49] define the total edge strength for pixel (x,y) in the image *I*

$$E\left(I\left(x,y\right)\right)=\max\left\{E_{1,3}\left(I\left(x,y\right)\right),E_{2,4}\left(I\left(x,y\right)\right)\right\}.$$

To keep the selected directions in a given pixel the same (consistent) for both compared images, the total edge strength for the second compared image *J* is defined by the formula:

$$E\left(J\left(x,y\right)\right)=\left\{\begin{array}{c} E_{1,3}\left(J\left(x,y\right)\right)\;\mathrm{if}\;E\left(I\left(x,y\right)\right)=E_{1,3}\left(I\left(x,y\right)\right)\\ E_{2,4}\left(J\left(x,y\right)\right)\;\mathrm{if}\;E\left(I\left(x,y\right)\right)=E_{2,4}\left(I\left(x,y\right)\right) \end{array}\right.$$

To compute the directional derivative of $\partial I_{j}\left(x,y\right)$, we use the convolution of the image function I(x,y) with the function $\omega_{j}\left(x,y\right)$ (see Equation (2)) that highlights the edges. The matrices of the convolution functions $\omega_{j}\left(x,y\right)$ for each direction j=1,2,3,4 are as follows:

$$\omega_{1}=\frac{1}{16}\left(\begin{array}{ccccc} 0 & 0 & 0 & 0 & 0\\ 0 & -3 & 0 & 3 & 0\\ 0 & -10 & 0 & 10 & 0\\ 0 & -3 & 0 & 3 & 0\\ 0 & 0 & 0 & 0 & 0 \end{array}\right),\;\omega_{2}=\frac{1}{16}\left(\begin{array}{ccccc} 0 & 0 & 3 & 0 & 0\\ 0 & 0 & 0 & 10 & 0\\ -3 & 0 & 0 & 0 & 3\\ 0 & -10 & 0 & 0 & 0\\ 0 & 0 & -3 & 0 & 0 \end{array}\right),$$

$$\omega_{3}=\frac{1}{16}\left(\begin{array}{ccccc} 0 & 0 & 0 & 0 & 0\\ 0 & 3 & 10 & 3 & 0\\ 0 & 0 & 0 & 0 & 0\\ 0 & -3 & -10 & -3 & 0\\ 0 & 0 & 0 & 0 & 0 \end{array}\right),\;\omega_{4}=\frac{1}{16}\left(\begin{array}{ccccc} 0 & 0 & 3 & 0 & 0\\ 0 & 10 & 0 & 0 & 0\\ 3 & 0 & 0 & 0 & -3\\ 0 & 0 & 0 & -10 & 0\\ 0 & 0 & -3 & 0 & 0 \end{array}\right).$$

The resulting value (index) of the similarity of the two images is expressed by the formula

(6) $$\mathrm{ESSIM}\left(I,J\right)=\frac{1}{MN}\sum_{x=0}^{M-1}\sum_{y=0}^{N-1}\frac{2E(I(x,y))E(J(x,y))+C}{E^{2}\left(I\left(x,y\right)\right)+E^{2}\left(J\left(x,y\right)\right)+C}.$$

Parameter *C* has a similar role to the SSIM measure and Zhang et al. [49] proposed to calculate it from the formula $C={\left(B\cdot L\right)}^{2p}$, where *B* is a predefined constant and *L* is range of pixel intensities of the compared images. For the standard 8-bit grayscale images is L=255.

In our experiments, we used the same values as suggested by the authors, i.e., B=0.1 and p=1.0 because these constants gave sufficiently good results.

### 4.4. Index Maps

The UIQI, SSIM, and ESSIM measures make it possible to visualize the differences between the compared images using so-called index (difference) maps. These difference maps are calculated from the local index values for each pixel (x,y), similar to the SSIM case where the local indices were ${\mathrm{SSIM}}_{xy}\left(I\left(x,y\right),J\left(x,y\right)\right)$. For better visualization, we added the power α in the difference map values for SSIM and ESSIM indices

(7) $${\mathrm{SSIM}}_{map}\left(I\left(x,y\right),J\left(x,y\right)\right)={\left({\mathrm{SSIM}}_{xy}\left(I\left(x,y\right),J\left(x,y\right)\right)\right)}^{\alpha }.$$

In our case, we chose α=1.2 for SSIM and α=1.5 for ESSIM. The UIQI measure did not provide good results, so we did not calculate difference maps for it.

### 4.5. Image Entropy

The last measure that we used in our calculations to compare the two images was the entropy of the image. The image entropy uses the histogram h(i), which is calculated from the pixel intensity values of the grayscale image and is expressed by the formula

(8) $$\mathrm{entropy}\left(I\right)=\sum_{i=0}^{255}p\left(i\right)\log_{2}p\left(i\right),\;\mathrm{where}\;p\left(i\right)\neq 0,\;\mathrm{for}\;p\left(i\right)=\frac{h(i)}{\sum_{i=0}^{255}h(i)}.$$

By calculating the image entropy for all scanning modes (see Table 1, Table 2, Table 3, Table 4, Table 5 and Table 6) of the three scanned objects, we found that the *LLa* scanning mode provides the “most information” in the image. The scan mode that would provide the lowest image entropy for all three scanned objects was not clearly determined. Therefore, we decided to use *LRFB* as a reference (ground truth) scanning mode, which excels in sufficiently homogeneous illumination of the entire scanned area of the object from all sides. In our experiments, we further compared all scan modes with the selected *LRFB* mode. Because the results of the 15-degree tilt scan were significantly different from the standard no-tilt scan, we divided the comparisons into two groups.

### 4.6. Difference Image and Edge Accentuation

At the end of the experiment, we attempted to visualize the differences between the *LRFB* reference scan mode and the other modes to see how the individual images scanned in the different modes differed. The simplest way was to create a difference image using ordinary difference

$$D_{IJ}\left(x,y\right)=\left|I(x,y)-J(x,y)\right|,\;\mathrm{for}\;x=0,1,\ldots ,M-1\;\mathrm{and}\;y=0,1,\ldots ,N-1.$$

The difference images did not have enough contrast, so we adjusted them by rescaling the intensity

$$D_{IJ}\left(x,y\right)=\frac{D_{IJ}\left(x,y\right)-D_{min}}{D_{max}-D_{min}},$$

where $D_{max}=\max\left\{D_{IJ}\left(x,y\right)\right\}$ and $D_{min}=\min\left\{D_{IJ}\left(x,y\right)\right\}$. We then reduced the gamma value from 1.0 to 0.5 and inverted the intensities of the adjusted difference image.

Since the *LLa* scanning mode provided images with the largest difference and entropy compared to the *LRFB* reference mode (see tables below), we decided to highlight the edges directly in the scanned images to see if, in addition to the edges of the objects, damaged parts (scratches, pinholes, cavities, etc.) would also be visible in the image. In this case, any available edge filter (Sobel, Canny, Prewitt, Roberts, Laplace, etc.) could be used, but we chose the simplest Roberts edge detector. We modified this procedure slightly by using a coefficient of k=4 to make the edges more visible. We applied the convolution function ω(x,y) to the image function I(x,y) and have

$$I^{\prime }\left(x,y\right)={\left(k\cdot (G_{x}^{2}\left(x,y\right)+G_{y}^{2}\left(x,y\right))\right)}^{1/2},$$

where

$$G_{x}\left(x,y\right)=\left(\omega_{x}*I\right)\left(x,y\right),\;\omega_{x}=\left(\begin{array}{cc} 1 & 0\\ 0 & -1 \end{array}\right),$$

and

$$G_{y}\left(x,y\right)=\left(\omega_{y}*I\right)\left(x,y\right),\;\omega_{y}=\left(\begin{array}{cc} 0 & 1\\ -1 & 0 \end{array}\right).$$

**Figure 12:**
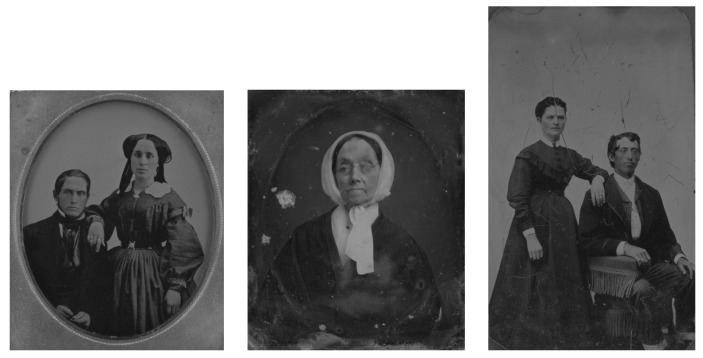
Grayscale images of ambrotype, daguerreotype, and tintype scanned in *LRFB* mode.

**Figure 13:**
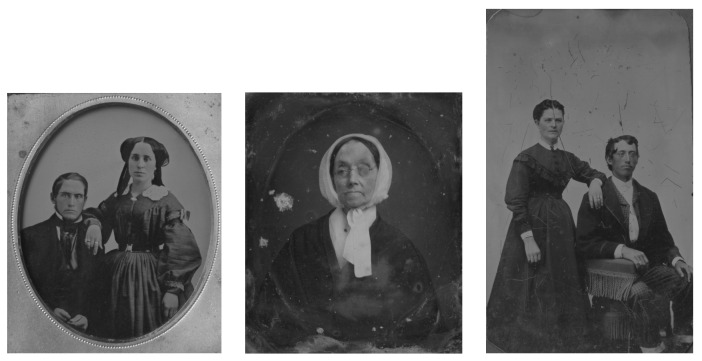
Grayscale images of ambrotype, daguerreotype, and tintype scanned in *LLa* mode.

**Figure 14:**
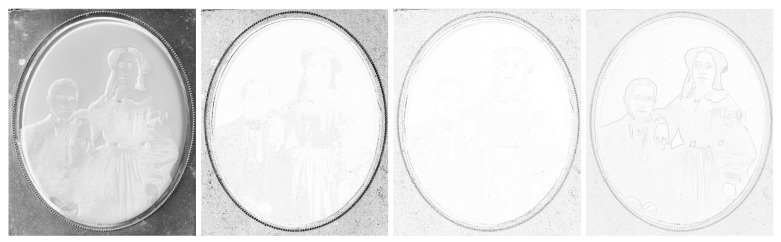
Difference images of ambrotype between *LLa* mode and *LRFB* reference mode. Left to right: difference image, SSIM map, ESSIM map, filtered edges of the image scanned in *LLa* mode.

**Figure 15:**
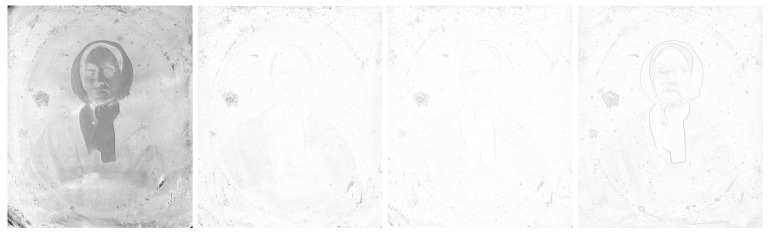
Difference images of daguerreotype between *LLa* mode and *LRFB* reference mode. Left to right: difference image, SSIM map, ESSIM map, filtered edges of the image scanned in *LLa* mode.

**Figure 16:**
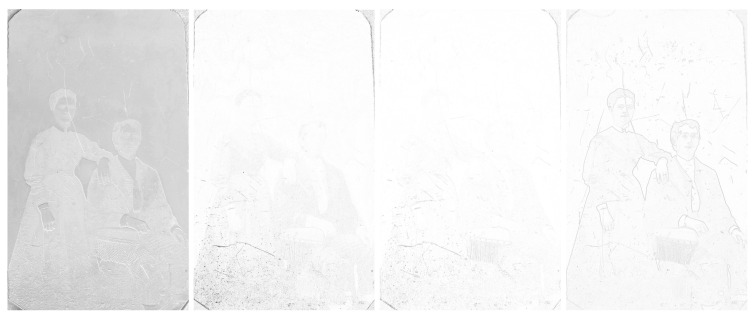
Difference images of tintype between *LLa* mode and *LRFB* reference mode. Left to right: difference image, SSIM map, ESSIM map, filtered edges of the image scanned in *LLa* mode.

We can see the grayscale Figure 12 and Figure 13, the adjusted difference images, difference maps and highlighted edges on images scanned in *LLa* mode in Figure 14, Figure 15 and Figure 16.

## 5. Results

In this section, we present the results we obtained by comparing images scanned in different scanning modes with images scanned in the *LRFB* reference mode. We used the UIQI, SSIM, and ESSIM measures described earlier. For each image, we also calculated the image entropy.

The results presented in the Table 1, Table 2, Table 3, Table 4, Table 5 and Table 6 show that *LLa* lighting mode generally provides the lowest similarity to *LRFB* lighting mode and has the highest image entropy. Values in bold indicate the lowest similarity to the image scanned in *LRFB* reference mode or the highest image entropy. Underlined values indicate the second-lowest similarity to the image scanned in *LRFB* reference mode or the second-highest image entropy. The largest entropy value in an image means that the image contains the most image information. This means that the surface damage of scanned objects is more visible in the *LLa* lighting mode than in the *LRFB* or other lighting modes. We can say that the shadows “added” by *LLa* mode are quite significant even when scanning flat objects. This information, together with difference images and difference maps, helps restorers locate damaged parts and determine the extent of surface damage to scanned objects. In conclusion, we can say that the *LLa* lighting mode is the best of all lighting modes when it is necessary to capture even minor damage to scanned objects.

## 6. Qualitative Evaluation of Selected Scans

Within this section, we cite the human expertise of experienced restoration professionals. They are responsible for the initial selection of three hard-to-photograph originals, the creation of the above-cited Europeana-style metadata, and the selection of the below commented DAG, AMB, and TIN scans or their processed versions in PNG image format. There are two levels of observation. First, the quality of the scan itself is for documentation and presentation. Second, the visual damage serves educational and possibly future restoration purposes.

### 6.1. Ambrotype

The grayscale image of ambrotype scanned with *LRFB* mode (see Figure 12 left): black and white flattened image of the hand-colored ambrotype allows us to look at the image as a whole without being disturbed by the coloring and the three-dimensionality of the image and glass surface. By viewing this image, we can focus more on the composition and tonality of the ambrotype.

The difference image of the *LRFB* vs. *LLa* scan mode (see Figure 14 left): does not give us much information needed for the correct identification or conservation treatment.

The SSIM map (see Figure 14 middle–left): does not give us much information needed for the correct identification or conservation treatment.

The ESSIM map (see Figure 14 middle–right): does not give us much information needed for the correct identification or conservation treatment.

The image of filtered edges of the picture scanned in *LLa* mode (see Figure 14 right): in this case, this type of rendering is useful mainly for showing the deterioration of the background of the image in form of little dots.

### 6.2. Daguerreotype

The grayscale image of daguerreotype scanned with *LRFB* mode (see Figure 12 middle): The difference image of the *LRFB* vs. *LLa* scan mode (see Figure 15 left): emphasize the imperfection of the daguerreotype (the plate) such as abrasions, scratches gouges, and corrosion.

The SSIM map (see Figure 15 middle–left): does not give us much information needed for the correct identification or conservation treatment.

The ESSIM map (see Figure 15 middle-right): does not give us much information needed for the correct identification or conservation treatment.

The image of filtered edges of the picture scanned in *LLa* mode (see Figure 15 right): all imperfections are here even more detailed visualized than on the Figure 15 left. It is hard to tell though if there are only imperfections of the daguerreotype plate showing here or also the small surface particles such as dust. This could be a little misleading at the end.

### 6.3. Tintype

The grayscale image of the tintype scanned with *LRFB* mode (see Figure 12 right): in the case of tintype, this rendering is not so much different from the original scan.

The difference image of the *LRFB* vs. *LLa* scan mode (see Figure 16 left): does not give us much information needed for the correct identification or conservation treatment.

The SSIM map (see Figure 16 middle–left): does not give us much information needed for the correct identification or conservation treatment.

The ESSIM map (see Figure 16 middle–right): does not give us much information needed for the correct identification or conservation treatment.

The image of filtered edges of the picture scanned in *LLa* mode (see Figure 16 right): this image is very helpful for determining the exact number of scratches in form of lines. It also shows the minor scratches on the photographs that otherwise might not be detected with the naked eye. Very helpful rendering in this case.

One can conclude that qualitative judgment offers support for rendering differences in addition to the primary look of a given digital twin of a cultural object. Moreover, there are indications for future research and the utility of tested methods for restoration practice.

## 7. Discussion

In general, we are aware of three types of uncertainty: vagueness (fuzziness), non-specificity, and controversy (strife). They cause certain experiences with scanning natural or cultural heritage items. In [22], we report on the missing data, limitations of sharpening or scanner precision, and lack of expertise when scanning such unusual originals, such as rare handmade papers for creative use in achieving real-media effects, resp., “ultra-realistic papers, canvases, and lithography stones developed in cooperation with academic professionals” [50]. The non-specificity of describing the original can be improved by clarifying metadata with respect to Europeana requirements or even to the international metadata standard CIDOC-CRM. The most challenging uncertainty seems to be the controversy. It is caused by multiple ground truths. Our InfoVis differentiation may help to support the decision-making in the time of scanning, and increase the accessibility of certain intuition for beginners or students, but this sort of machine perception does not suffice for restoration experts.

Our results can be seen in methodology, original problem statement, and workflow design. In particular, we contributed to the study of image formation with manual focusing enabled by CRUSE technology and postprocessing inspired by image quality methods and machine perception (feature points, novel coSIFT, evaluating SSIM, saliency, and other image quality properties). Last but not least, we confronted the automated processing with the perceptual experiment in the eye-tracking case study. Combining CRUSE scans with eye-tracking evaluation seems to be novel and not available in the given literature. The meaning of our approach can either influence the deeper study of already scanned and published images or even help to propose better or faster procedures in future digitization.

## 8. Conclusions

Our novel workflow for relatively systematic documenting of rare cultural objects can be characterized as a virtual museum creation. First, we chose the globally unique primary dataset of cultural objects. Second, we produced the secondary digital dataset. Third, we designed the scenarios for the presentation of their properties and comparisons. Fourth, we planned and implemented the integrated hardware and software solution. Fifth, we designed the scenarios for presentation, e.g., the web page [9] or this paper. Sixth, we integrated, verified, and tested our “virtual museum or exhibition”. Seventh, we publish and evaluate our findings, contribution, and data for target groups, resp. interests, including students at our faculties. As research on the CRUSE scanner properties is in its beginnings, we omitted previous work surveys, and except for the sharpening and coSIFT parts, we just mentioned relevant ideas at the appropriate places in the text. The sharpening or manual focusing, coSIFT, and scanning mode comparisons are systematic, but certain parts, such as heatmap comparisons are, obviously, in the proof-of-concept state.

Our experiments with image similarity and entropy measures, as well as evaluations by photo restoration experts, confirmed that among all lighting modes, *LLa* is the most advantageous. In some cases, differential images and differential maps are also useful, helping to visualize even very subtle surface deterioration that is not visible at first glance.

Our methodological and practical contributions imply several future research avenues (e.g., precision, perception, presentation, statistic evaluation, and appropriate visualization), which we support via the published dataset [9] under CC BY-NC 4.0 license.

## Figures and Tables

**Table 1:** Ambrotype. Comparison of images scanned in different scan modes with a reference image scanned in *LRFB* mode.

Ambrotype	UIQI	SSIM	ESSIM	Entropy
LRFB	1.0000	1.0000	1.0000	6.4305
LR	0.9903	0.9879	0.9962	6.4254
LFB	0.9881	0.9683	0.9887	6.6589
LF	0.9240	0.9573	0.9851	6.6550
LTx	0.9996	0.9441	0.9818	7.0454
LTx5cm	0.9903	0.9634	0.9875	6.6757
LTx10cm	0.9892	0.9667	0.9872	6.4510
RTx	0.9567	0.9453	0.9819	6.7601
RTx5cm	0.9881	0.9608	0.9852	6.5538
RTx10cm	0.9875	0.9643	0.9852	6.4417
LLa	0.9776	**0.9162**	**0.9743**	**7.0590**

**Table 2:** Ambrotype 15°. Comparison of images scanned in different scan modes with a reference image scanned in *LRFB* 15° mode.

Ambrotype 15°	UIQI	SSIM	eSSIM	Entropy
LRFB15deg	1.0000	1.0000	1.0000	6.9887
LR15deg	0.9721	0.9879	0.9963	6.9889
LFB15deg	0.9738	0.9680	0.9887	7.1650
LF15deg	0.9749	**0.9649**	0.9875	7.1690
L15deg	0.9993	0.9660	**0.9874**	**7.1763**

**Table 3:** Daguerreotype. Comparison of images scanned in different scan modes with a reference image scanned in *LRFB* mode.

Daguerreotype	UIQI	SSIM	ESSIM	Entropy
LRFB	1.0000	1.0000	1.0000	5.9694
LR	0.9969	0.9846	0.9951	5.9905
LFB	0.9993	0.9886	0.9965	5.9219
LF	0.9797	0.9871	0.9956	5.8905
LTx	0.9980	0.9858	0.9947	6.0465
LTx5cm	0.9979	0.9841	0.9946	5.9017
LTx10cm	0.9988	**0.9704**	**0.9899**	5.8276
RTx	0.9966	0.9844	0.9944	6.0112
RTx5cm	0.9986	0.9850	0.9952	5.9424
RTx10cm	0.9989	0.9806	0.9938	5.8953
LLa	0.9932	0.9793	0.9918	**6.0670**

**Table 4:** Daguerreotype 15°. Comparison of images scanned in different scan modes with a reference image scanned in *LRFB* 15° mode.

Daguerreotype 15°	UIQI	SSIM	eSSIM	Entropy
LRFB15deg	1.0000	1.0000	1.0000	6.0504
LR15deg	0.9930	**0.9843**	0.9948	6.0094
LFB15deg	0.9887	0.9873	0.9956	6.0540
LF15deg	0.9865	0.9868	0.9953	6.0584
L15deg	0.9952	0.9847	**0.9944**	**6.1109**

**Table 5:** Tintype. Comparison of images scanned in different scan modes with a reference image scanned in *LRFB* mode.

Tintype	UIQI	SSIM	eSSIM	Entropy
LRFB	1.0000	1.0000	1.0000	5.5269
LR	0.9969	0.9783	0.9928	5.5345
LFB	0.9995	0.9801	0.9930	5.5269
LF	0.9738	0.9817	0.9937	5.5560
LTx	0.9996	0.9792	0.9929	5.6443
LTx5cm	0.9973	0.9804	0.9935	5.5671
LTx10cm	0.9970	0.9784	0.9931	5.4833
RTx	0.9932	0.9797	0.9928	5.5510
RTx5cm	0.9983	0.9816	0.9938	5.4741
RTx10cm	0.9992	0.9785	0.9929	5.4091
LLa	0.9984	**0.9767**	**0.9920**	**5.7240**

**Table 6:** Tintype 15°. Comparison of images scanned in different scan modes with a reference image scanned in *LRFB* 15° mode.

Tintype 15°	UIQI	SSIM	eSSIM	Entropy
LRFB15deg	1.0000	1.0000	1.0000	5.5559
LR15deg	0.9888	0.9844	0.9949	5.5757
LFB15deg	0.9889	0.9840	0.9946	5.6118
LF15deg	0.9891	**0.9824**	**0.9938**	5.6180
L15deg	0.9997	0.9837	0.9944	**5.6524**

## Data Availability

The data supporting the reported results can be found at [9], available under the CC BY-NC 4.0 license.

## Abbreviations

The following abbreviations are used in this manuscript:

AFAD	Academy of Fine Arts and Design
GAN	generative adversarial network
RNG	relative neighborhood graph
MST	Minimum Spanning Tree
AMB	ambrotypes
DAG	daguerreotypes
TIN	tintypes
DT	Delaunay triangulation
SIFT	stop, investigate, find, and trace
DFT	discrete Fourier transform
DCT	discrete cosine transform
PPI	pixels per inch
HVS	human visual system
MSE	mean square error
RMSE	root mean square error
UIQI	universal image quality index
SSIM	structural similarity index measure
ESSIM	edge strength similarity image quality metrics
